# NUT Carcinoma—An Underdiagnosed Malignancy

**DOI:** 10.3389/fonc.2022.914031

**Published:** 2022-07-26

**Authors:** Ulrich M. Lauer, Martina Hinterleitner, Marius Horger, Paul V. Ohnesorge, Lars Zender

**Affiliations:** ^1^ Medical Oncology and Pneumology, Internal Medicine VIII, University Hospital Tübingen, Tübingen, Germany; ^2^ German Cancer Research Center (DKFZ), German Cancer Consortium (DKTK), Tübingen, Germany; ^3^ Cluster of Excellence iFIT (EXC 2180) “Image-Guided and Functionally Instructed Tumor Therapies”, University of Tübingen, Tübingen, Germany; ^4^ Department of Diagnostic and Interventional Radiology, Eberhard Karls University, Tübingen, Germany

**Keywords:** NUT carcinoma, NUTM1, NUT rearrangement, BET inhibitor, BRD4

## Abstract

NUT carcinoma (NC) is a rare and highly aggressive malignancy with a dismal prognosis and a median survival of 6–9 months only. Although very few cases of NC are reported each year, the true prevalence is estimated to be much higher, with NC potentially widely underdiagnosed due to the lack of awareness. NC primarily occurs in midline structures including thorax, head, and neck; however, other sites such as pancreas and kidney are also affected, albeit at lower frequencies. NC is characterized by a single translocation involving the *NUTM1* (NUT midline carcinoma family member 1) gene and different partner genes. The resulting fusion proteins initiate tumorigenesis through a mechanism involving BET (bromo-domain and extra-terminal motif) proteins such as Bromodomain-containing protein 4 (BRD4) and inordinate acetylation of chromatin, leading to the dysregulation of growth and differentiation genes. While no clinical characteristics are specific for NC, some histologic features can be indicative; therefore, patients with these tumor characteristics should be routinely tested for NUTM1. The diagnosis of NC using immunohistochemistry with a highly specific antibody is straightforward. There are currently no standard-of-care treatment options for patients with NC. However, novel therapies specifically addressing the unique tumorigenic mechanism are under investigation, including BET inhibitors. This review aims to raise awareness of this underdiagnosed cancer entity and provide all patients the opportunity to be properly diagnosed and referred to a clinical study.

## Introduction

NUT carcinoma (NC) is a rare and aggressive subtype of squamous carcinoma characterized by genetic rearrangements involving the *NUTM1* (NUT midline carcinoma family member 1) gene. Patient cases with tumors harboring a t(15;19) translocation were reported from 1991 onwards (1–3). In 2003, the fusion gene resulting from this translocation was identified as *BRD4-NUT*. Many other fusion partners have been identified since, including BRD3, NSD3, ZNF532, ZNF592, and CIC (4–7), leading to the notion that classification, clinical behavior, and therapeutic options may be best defined by the NUTM1 fusion partner rather than by tumor morphology or the immunohistochemical profile (8, 9).

NC affects people of any age. The actual incidence of NC is unknown; however, only ~300 cases have been reported in the literature to date. Most of those cases were based in the United States, where NC has been intensively studied, suggesting that awareness in other countries may be low and the diagnosis often missed. Recently, the incidence of NC could be determined for the first time in the geographically isolated state of Western Australia with a well-defined pediatric population, determining an estimated incidence of NC of approximately 0.41 per million child years (0–16 years of age) at risk (10).

Although NCs often arise from midline structures such as thorax or head and neck (thereby leading to the nomenclature “NUT midline carcinoma”), NC can originate in almost any body site, for example in the kidneys or in pancreas (11, 12). At present, it is unclear from which cell type NC originates, as patients are frequently diagnosed at an advanced stage of disease, often with invasive and metastatic tumors. Frequently, the primary tumor site cannot be identified, and cases of CUP (cancer of unknown primary) syndrome, which accounts for up to 5% of malignant diseases, may in fact represent NUT carcinomas (13). In such instances, it is tempting to speculate an aberrant germ cell phenotype as the origin of NCs (14). Suspected cases should therefore undergo further investigation to improve the success of differential diagnosis.

There is currently no standard-of-care therapy for NC, and patients face a grim prognosis, with a median survival of 6–9 months only (15, 16). Approximately 70%–80% of diagnosed patients succumb to the disease within 2 years (15–17), although some long-term survivors have been reported (18–20). Because the malignancy is rare, little is known so far about the risk profiles associated with the diverse tumor origins and heterogeneous *NUTM1* fusion partners. Accordingly, contributions to the International NC Registry (see below) are highly needed.

In this review, we aim to summarize current knowledge about the origins, diagnosis, and treatment of NC; provide insight into ongoing efforts to raise the awareness of this underdiagnosed lethal disease; and encourage the development of effective therapeutic options.

## Prevalence and Demographics

NC is a rare malignancy and the true prevalence is not known, possibly due to an absence of the reporting of diagnosed cases. The International NUT Midline Carcinoma Registry (http://www.nmcregistry.org), which provides information and guidance around NC recognition and treatment, reports approximately 20 new cases per year (21); however, we assume that not all diagnosed cases are submitted to the Registry.

**Figure 1:**
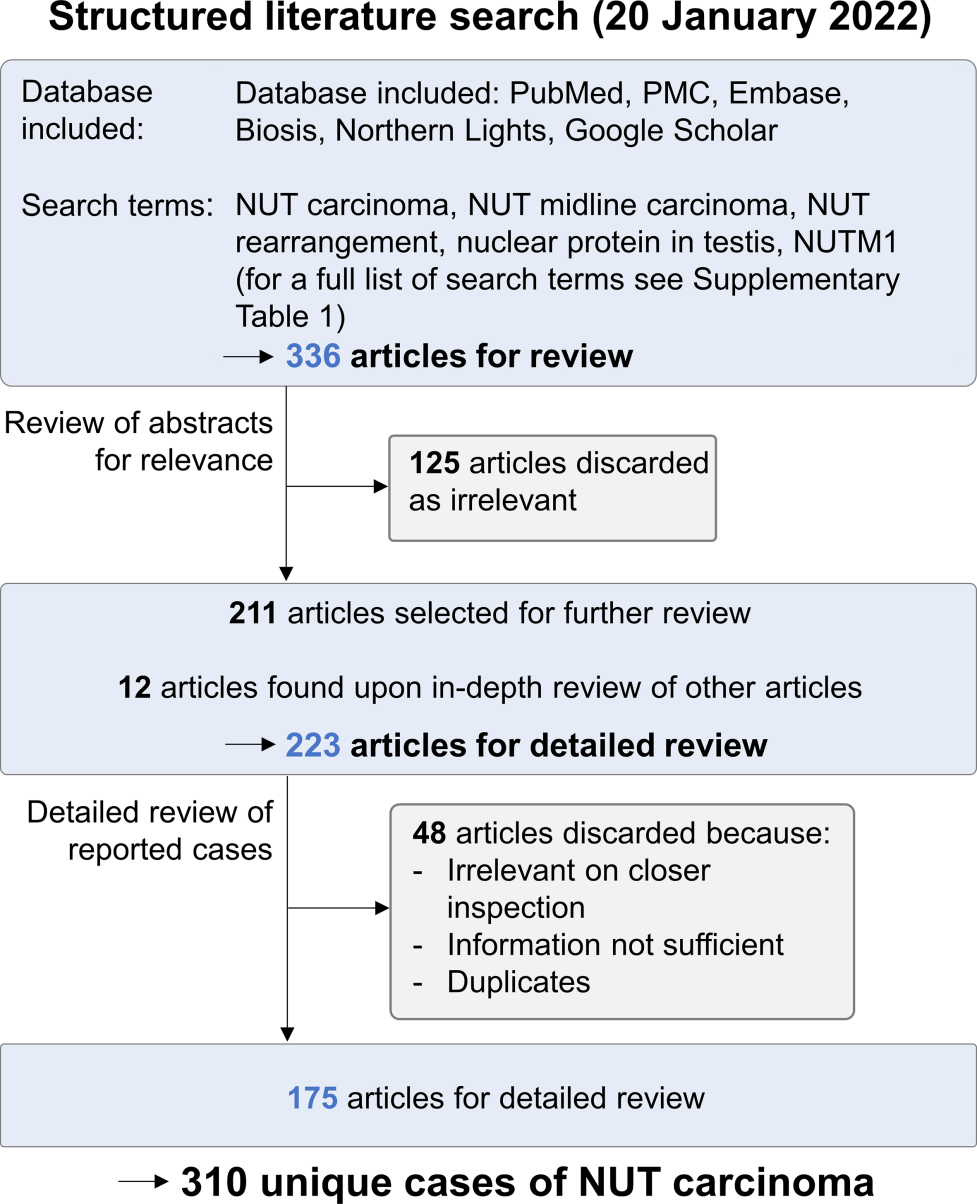
Structured literature search for NC case reports. A structured literature search for published cases of NC was performed on January 20, 2022. Of 336 cases retrieved, 125 were discarded as irrelevant upon the analysis of abstracts. Upon further review of the remaining articles, 12 additional publications were identified. All 223 articles were reviewed in detail; 48 were discarded because they were irrelevant on closer review, did not provide sufficient information on patient data, or contained duplicate cases. The remaining 175 articles provided data on 310 unique cases of NC.

**Figure 2:**
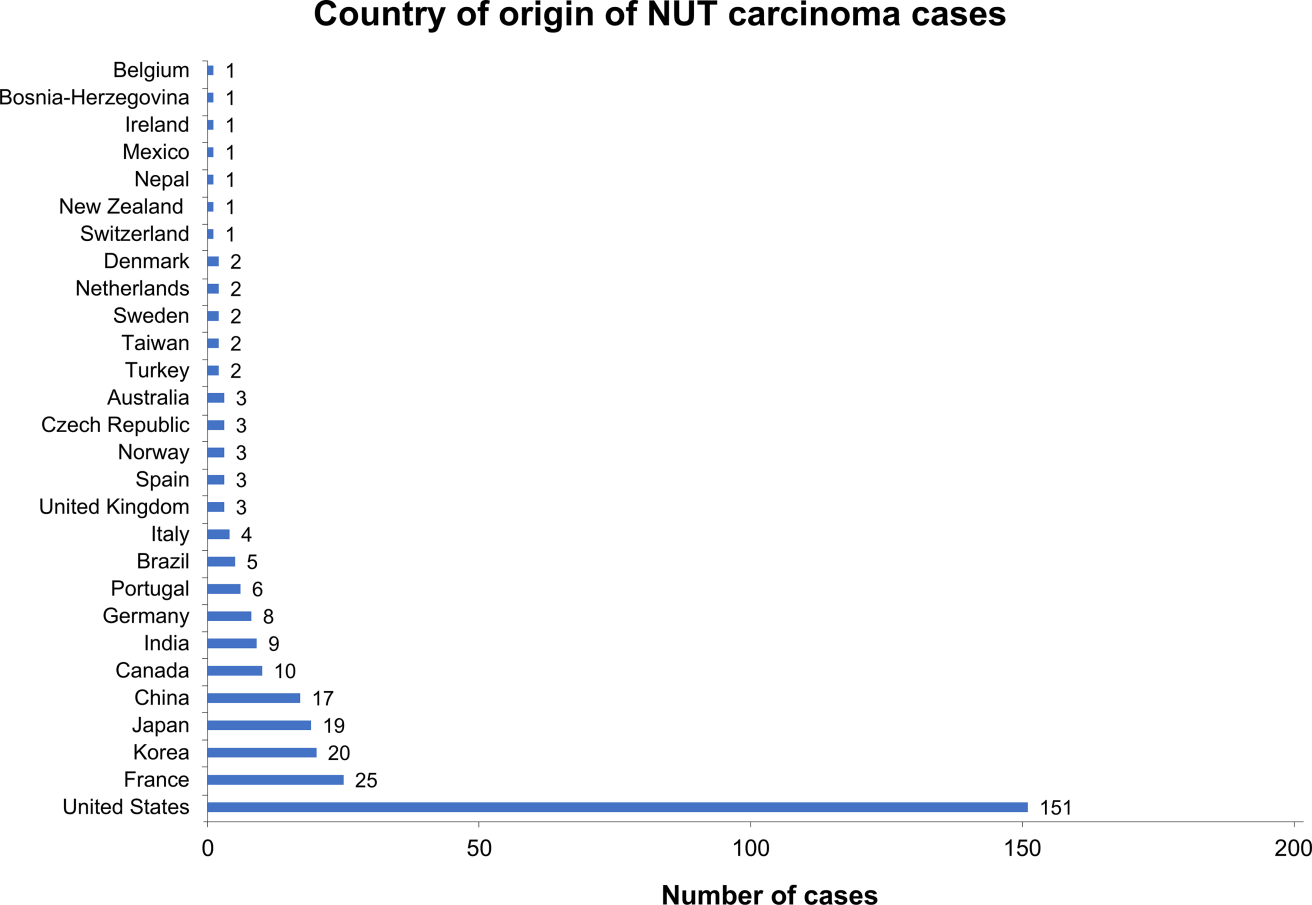
Country of origin of NC cases that were identified in structured literature search. For 306 of the 310 identified cases, the country of origin was available. About half of these presented in the United States, whereas other countries such as Germany were underrepresented relative to the overall population size, suggesting that NC is underdiagnosed.

In a structured literature analysis to identify NC case reports, we identified 310 unique cases published since 1991 (**Figure 1**). Of note, 151 of these (49%) were based in the United States (**Figure 2**). By comparison, only 8 cases were reported in Germany, implying a reporting bias, not only potentially due to a lack of awareness or misdiagnosis, but also possibly due to a lesser tendency to publish diagnosed cases. The age at diagnosis was available for 308 patients and showed a wide range from 0 (newborn) to 82 years, demonstrating that NC affects people of every age; the median age at diagnosis was 30 years. Sex distribution was balanced, with 57% of the cases being male patients and 43% female. Information about gender was not reported for 8 cases. Due to differences in description, a wide range of primary tumor sites was reported; however, common sites included the lung (n=87), mediastinum (n=44), nose/nasal cavity (n=31), sinus/sinunasal tract (n=19), parotid gland (n=9), hilum (n=7), and maxilla/maxillary gland (n=7). Death was reported in 165 cases. The median survival from diagnosis to death was 5.5 months (range 0.09–48 months), reflecting a highly aggressive malignancy (15–17).

These data suggest that NC remains highly underdiagnosed and/or under-reported, especially among adults, in most regions of the world and the true prevalence may be much higher than the literature record implies.

## Molecular Genetics and Tumor Transformation

Unlike other solid tumors with complex chromosomal rearrangements and a high mutational burden, NCs display a simple karyotype, often with only one alteration comprising the translocation that fuses *NUTM1* to one of its partners. A recent sequencing approach supports the finding that NCs harbor a single driver mutation and that the NUTM1 fusion protein is sufficient for the oncogenic transformation of hitherto healthy dividing cells (22).

The genetic hallmark of NUT carcinomas is a translocation of the *NUTM1* [formerly known as *NUT* (nuclear protein in testis) gene]. The most common *NUTM1* fusion partner is *BRD4* (bromo-domain-containing protein 4), a ubiquitously expressed transcriptional activator (15, 23). The resulting fusion protein comprises the complete coding region of NUTM1 fused to all but one functional domains of BRD4, including the two bromo-domains that bind acetylated lysine residues (7). The *BRD4-NUTM1* fusion gene is driven by the *BRD4* promoter.

NUTM1 is a rather unstructured protein of unknown function (6). Aside from a nuclear localization signal and a nuclear export signal, NUTM1 harbors two acidic domains, one of which has been shown to bind the histone acetyl transferase (HAT) EP300 (21). BRD4 belongs to the BET (bromo-domain and extra-terminal motif) protein family and is known to bind acetylated histones in transcriptionally active chromatin (24).

*BRD4* was the *NUTM1* fusion partner in 75% of NC cases identified in the literature search for which genetic information was reported (**Supplementary Figure 1**). Other *NUTM1* fusion partners that have been described include *BRD3*, *NSD3*, *ZNF532*, *ZNF592*, and *CIC*. While BRD3 has a predicted function similar to BRD4, the other genes encode proteins with diverse functions. However, BRD3, NSD3, and ZNF532 have all been shown to bind BRD4, and the corresponding NUTM1 fusions appear to provoke a common mechanism in a potent oncogenic protein complex (4, 5, 23).

**Figure 3:**
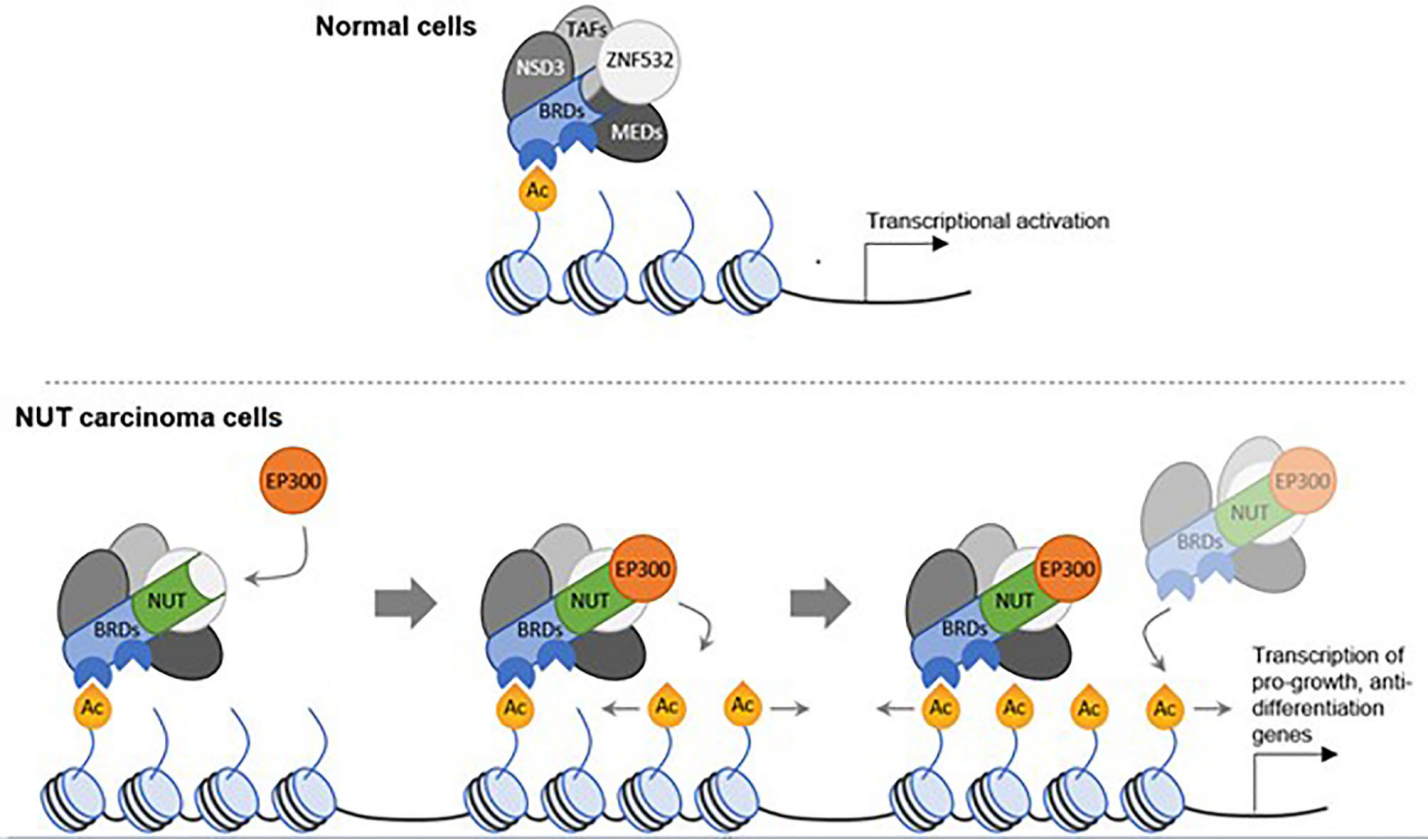
A model for tumor development in NC through oncogenic mega-domain formation. In healthy cells, BRD complexes including transcription activation factors and mediator complex subunits serve as transcriptional facilitators. These complexes comprise *NSD3* and *ZNF532*. In NC cells, a single translocation creates a *NUTM1* fusion protein, in most cases with *BRD4* or *BRD3* as the fusion partner. The bromo-domains of the BRD proteins tether *NUTM1* to acetylated chromatin. *NUTM1*, in turn, recruits the histone acetyl transferase *EP300*, thereby increasing the acetylation of the surrounding chromatin. In a feed-forward mechanism, more NUT-fusion protein complexes bind to the additional acetylation sites and the increased acetylation expands, creating acetylated mega-domains in which proliferation genes are transcribed. (23, 25).

In a postulated mechanism of action, the BRD4-NUTM1 fusion protein tethers NUTM1 to acetylated chromatin through the BRD4 bromo-domains. As a result, HAT EP300 is recruited to these chromatin sites by binding to NUTM1, which leads to the acetylation of the surrounding chromatin and formation of large stretches of active chromatin, termed mega-domains (**Figure 3**). Transcriptional activators are thereby recruited, and genes that promote cellular growth, such as *MYC* and *TP63*, are expressed, while pro-differentiation genes outside the mega-domains are suppressed (21, 25).

## Imaging and Histology

NCs are heterogeneous in appearance, and routine imaging techniques reveal no specific distinguishing features. Unfortunately, NCs are often aggressive, with a tendency to invade neighboring structures (26) or form necrotic metastatic lesions (27). Although these features are characteristic of squamous cell carcinomas, these cancers are unusual in children and adolescents; therefore, NC should be considered when these features are observed, especially in young patients (28).

For correct staging of NCs, contrast-enhanced computed tomography is generally the recommended approach. Magnetic resonance imaging can give additional information, as this technique facilitates a thorough evaluation of soft tissue, especially when assessing possible bone and/or vascular infiltration or when analyzing the head and neck region (29). Fludeoxyglucose positron emission tomography can help to identify distant metastases, although fludeoxyglucose accumulates preferentially in non-necrotic masses and the low signal in necrotic areas may lead to an underestimation of disease burden (30).

**Figure 4:**
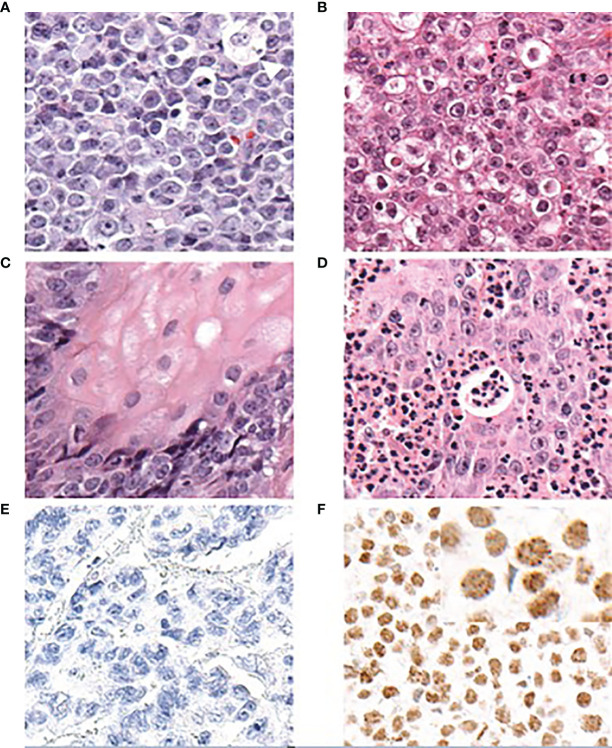
Diagnosis of NC based on histopathological features and IHC to *NUTM1* (antibody clone C52B1 by Cell Signaling Technology, Danvers, MA, USA) (adapted from French et al., 2018, and reprinted with permission). **(A)** NCs typically show a monomorphic appearance with round cells. The white arrowhead indicates a cell with “fried-egg” appearance. **(B)** In certain areas, altered cells may appear more frequently. **(C)** In some but not all NC tumor samples, focal squamous differentiation can be observed. **(D)** A common feature is the (sometimes prominent) infiltration by neutrophilic cells. **(E)** Non-NC tumor tissue does not show any staining of *NUTM1*. **(F)** Nuclei in NC tumor tissue show diffuse staining with a typically speckled appearance. A positive staining for *NUTM1* in more than 50% of nuclei in a tumor sample is diagnostic for NC. NUT Carcinoma: Clinicopathologic features, pathogenesis, and treatment, Christopher A. French. Copyright © 2018 Japanese Society of Pathology and John Wiley & Sons Australia, Ltd. Reproduced with permission of John Wiley & Sons Ltd.

The histopathological analyses of NCs have failed to identify a unique morphology of the diagnostic value. NCs show poor overall differentiation and can be mistaken for other undifferentiated tumors, including other poorly differentiated squamous cell carcinomas or Ewing sarcoma (18, 31, 32). A few histopathological features, however, are fairly characteristic of NC, including a distinctive monomorphism of small-to-medium sized cells, distinguishing NC from other poorly differentiated tumors, which often have a more pleiotropic appearance (**Figure 4A**) (21).

The chromatin of NC cells is generally vesicular, often with numerous cells showing a clear cytoplasm that resembles fried eggs (**Figure 4B**). In up to 40% of histologic samples, the foci of abrupt squamous differentiation can be observed (**Figure 4C**) (15, 17). Another common feature of NC tumor tissue is the infiltration of neutrophils (**Figure 4D**) (23).

Taken together, there are some features that are suggestive of NC when observed in a tissue sample, although they are not always present and not singular enough to precisely diagnose NC. These features can, however, be used to systematically include NC in a diagnostic scheme to ensure that NC is at least considered in any patient presenting with these characteristics.

## Diagnosis

NC is characterized by its aggressive growth. Patients often present with advanced or metastatic disease, and the primary tumor site often remains undetermined.

Because no morphological features are truly unique to NC, it is diagnosed solely on the basis of its hallmark genetic characteristic, the *NUTM1* rearrangement that results in the expression of an NUTM1 fusion protein. Under normal circumstances, the expression of wild-type NUTM1 is restricted to the testes and any expression outside the testes (or a germ cell tumor) can be interpreted as the pathologic expression of an *NUTM1* fusion gene driven by the promoter of its fusion partner. A diagnosis of NC can be achieved using immunohistochemical (IHC) staining and a specific monoclonal antibody that recognizes the NUTM1 protein (clone C52B1, Cell Signaling Technology, Danvers, MA, USA) (**Figures 4E, F** **)** (33). IHC staining using this antibody is 100% specific and shows a sensitivity of 87% for a diagnosis of NC when more than 50% of nuclei on formalin-fixed, paraffin-embedded tissue sections are stained (33). Therefore, this simple pathological test is generally considered sufficient to diagnose NC tumor tissue, as affirmed by the World Health Organization (WHO) classification of tumors (34).

Additional tests can be performed to identify the fusion partner of *NUTM1*. This might be of clinical and prognostic relevance, as, for example, *NSD3* and *BRD3* as fusion partners have been associated with better overall survival (OS) than *BRD4*, at least for the tumors of non-thoracic origin (17). Diagnostic confirmation can be achieved with fluorescence *in situ* hybridization (FISH) using split-apart probes (35); other approaches include next-generation sequencing (NGS)-based assays such as RNA sequencing (4, 5) or Archer^®^ FusionPlex^®^ (ArcherDX, Boulder, CO, USA) (36).

While a diagnosis of NC is not complex once investigated properly, a larger hurdle appears to be a widespread lack of awareness of this rare disease and consideration, in the presence of suggestive features, of NC as a possible diagnosis.

NCs often arise from midline upper airway locations or the mediastinum (37), an observation that gave the disease its original name “NUT midline carcinoma.” However, as mentioned, many other sites of origin such as kidney, pancreas, or bone, have been described and a tumor location outside the midline definitely should not exclude NC as a diagnosis (11, 12, 18).

Men and women are affected equally by NC, and risk factors such as smoking are currently not known to be relevant for the development of NC (23). Therefore, neither age nor sex, the tissue of primary malignancy, or history of smoking can be used to exclude NC.

A virus-related etiology, such as Epstein–Barr virus or human papilloma virus, has not been reported in association with NCs (23). Similarly, glandular differentiation is not common in NC; therefore, upon the observation of glandular differentiation, tumors need not be tested for NC.

**Figure 5:**
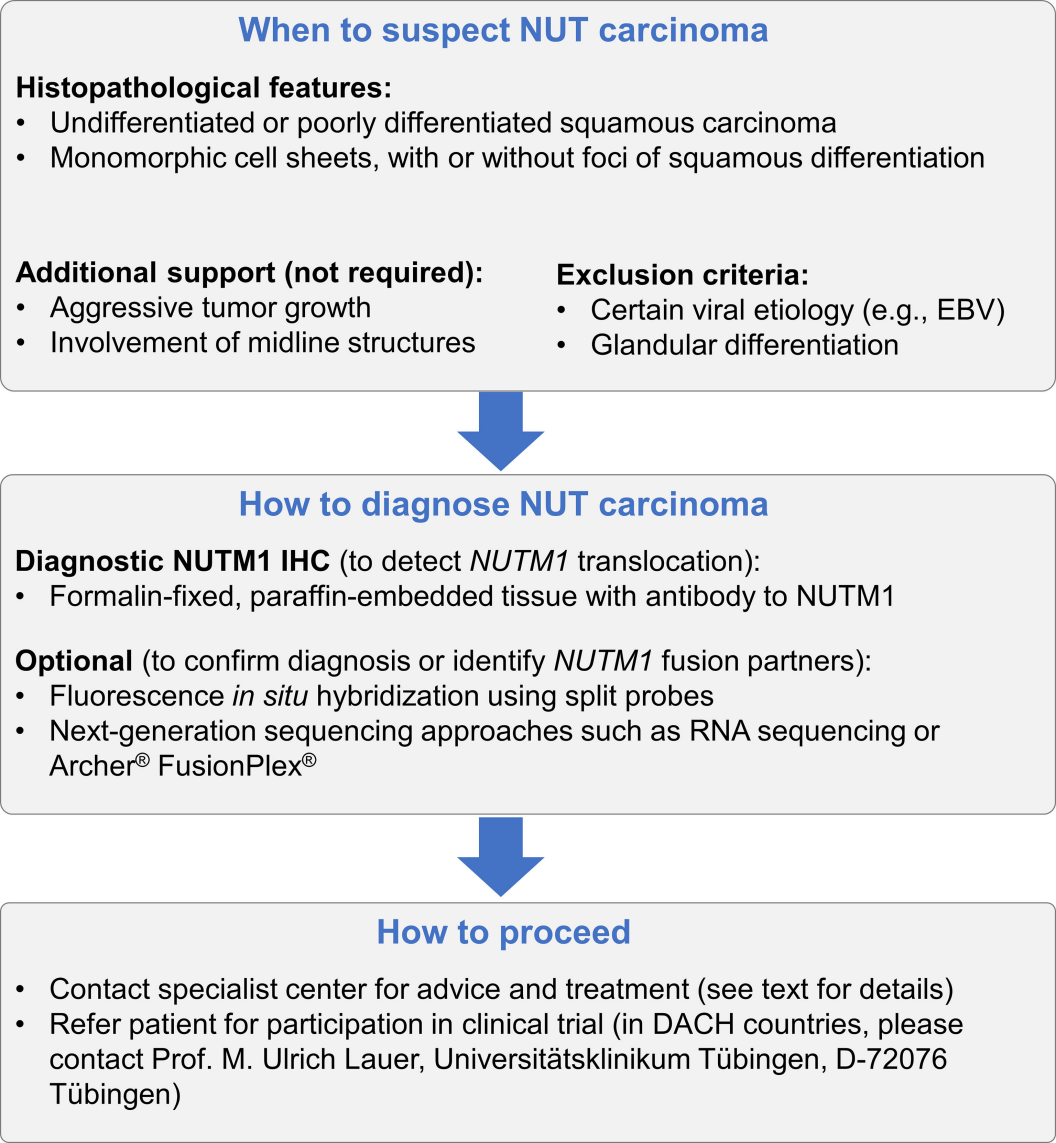
Recommended algorithm to ensure that a diagnosis of NC is considered in appropriate patients. NUT carcinoma should be considered in every patient presenting with undifferentiated or poorly differentiated squamous carcinoma with monomorphic cells, regardless of the presence of the foci of squamous differentiation. NCs are often characterized by aggressive growth and often, although not always, originate from midline structures. Viral etiology has not been associated with NC, and glandular differentiation is not common; therefore, both can be used to exclude NC. For the diagnosis of NC, it is sufficient to demonstrate the expression of NUTM1 in tumor tissue using IHC to *NUTM1.* Additional approaches can be chosen to validate the diagnosis or to identify the fusion partner of NUTM1.

The possibility of an NC malignancy should be considered in cases where metastases are present, although the primary tumor is unknown. A diagnosis of NC should be considered and tested for in all poorly differentiated, non-cutaneous carcinomas with a monomorphic appearance, with or without local squamous differentiation (**Figure 5**).

## Treatment

Given its rarity and heterogeneity, there is currently no effective standard therapy for patients diagnosed with NC, and the general prognosis is daunting. Once diagnosed, patients are often treated with a combination of surgery, radiation, and chemotherapy. Some small studies have shown successful outcomes with this multimodal approach. For example, two pediatric cases with the NC of the head and neck achieved a complete response (one with a duration of >6 years) (38). However, complete surgical resection is often not possible, and even when possible, patients usually experience rapid disease progression following a complete response to initial treatment and adjuvant chemotherapy and/or radiation. For example, in a single-center study of five pediatric patients with head and neck NC, outcomes with aggressive multimodal treatment were poor, with median progression-free survival (PFS) of 3 months and median OS of 10 months (39). Similarly, in a case of 10 patients with the NC of the parotid, sublingual or submandibular glands, outcomes with multimodal treatment were disappointing with an OS of only 5.5 months (40). It is clear, therefore, that novel targeted strategies for NC are an unmet medical need. Nevertheless, upfront surgical resection should be performed whenever feasible, as it seems to be associated with improved PFS and OS compared with radiotherapy ± chemotherapy or chemotherapy alone (15, 16). Of note, patients in the NC registry with thoracic NC also had worse prognosis than those with non-thoracic NC (17), regardless of tumor genetics, possibly due to the surgical challenges associated with accessing thoracic tumors.

Different systemic strategies have been reported, most commonly comprising platinum-based chemotherapies, either alone or in combination with other agents. An initial response to treatment is regularly observed; however, this approach is often of limited value and rapid disease progression is common (15). This reflects the fact that standardized chemotherapy regimens are currently not available; multiple chemotherapy compounds have been used in this setting including cisplatin, carboplatin, cyclophosphamide, doxorubicin, actinomycin D, etoposide, vinorelbine, vinblastine, vincristine, paclitaxel, docetaxel, bleomycin, ifosfamide, 5-fluorouracil, S-1, and gemcitabine (41). Isolated cases of long-term survival have been reported in patients treated with the Scandinavian Sarcoma Group’s SSG IX protocol developed for Ewing’s sarcoma (18, 38, 42); however, treatment failures using this protocol have also been reported. Because NC is rare and patient numbers are small, a statistical evaluation of different treatment strategies is challenging and, at present, no chemotherapeutic regimen can be recommended over other schemes.

With the discovery of the oncogenic driver mechanism, NC has become a promising candidate for targeted therapeutic strategies. One strategy is to use histone deacetylase inhibitors (HDACis) to reduce the hyperacetylated chromatin in the BRD4 mega-domains, thus restoring cellular transcription to normal levels (43). Promising results have been reported with HDACis in patient case studies (44, 45) and a phase I study is investigating the safety and preliminary anti-cancer activity of the HDACi (and PI3K inhibitor), CUDC-907 (NCT02307240, recruitment completed in 2019) (46). Since positive studies are still missing, HDACi treatment currently cannot be recommended as a standard of care in NC patients.

Another approach is focused on BET inhibitors (BETis), which mimic acetylated histones and competitively inhibit the binding of BET proteins such as BRD4 to acetylated chromatin (47). Initial promising results were recently reported for patients treated with the BETi birabresib (MK-8628/OTX015) (48). In this phase Ib study, a recommended phase II dose (RP2D) of 80 mg once daily on a continuous schedule was established. Notably, treatment with birabresib was found to be tolerable; the most common treatment-related adverse events were diarrhea (37%), nausea (37%), anorexia (30%), vomiting (26%), and thrombocytopenia (22%). Only three patients (7%) had to discontinue treatment due to adverse events. Encouragingly, three patients (33%) with NC had a partial response (duration: 1.4–8.4 months). No responses were observed in patients with other tumor types.

Another BETi agent, molibresib (GSK525762), has also recently been assessed in patients with NC and other cancers (49, 50). In the phase I part of a phase I/II study, molibresib was well tolerated with a similar safety profile as birabresib, characterized by gastrointestinal events (22%–42%) and thrombocytopenia (51%) (49). The RP2D was 80 mg once daily. Of 19 patients with NC, 4 (21%) achieved a partial response (confirmed or unconfirmed). In the phase II part of the study, only one patient with NC achieved a partial response and the predefined clinically meaningful response rate was not achieved (50). Nevertheless, the authors concluded that the assessment of combinatorial approaches based on BET inhibition are warranted. Molibresib has also been assessed in selected patients in an expanded-access study (NCT03702036) (51). The BETi compound RO6870810 has also been assessed in a phase I study of patients with NC and other cancers (52). Two of eight patients (25%) with NC responded to treatment.

Another BETi drug, BI 894999, has been under investigation in a recent phase I clinical trial in patients with advanced malignancies including NC (NCT02516553) (53). This study reached its completion date on Nov 23, 2021; the publication of results is expected in 2022. Preliminary data from the study demonstrated that BI 894999 was tolerable and demonstrated target engagement. The R2PD dose was 2.5 mg/day (2 weeks on/1 week off). There were preliminary signs of clinical activity, with partial responses observed in patients with sinus adenocarcinoma, squamous cell carcinoma of the esophagus, and jejunum adenocarcinoma, although no data in patients with NC have been published yet (54).

Another BETi, BMS-986158, is currently being evaluated in pediatric/juvenile cancers (aged 1–21 years) including those with translocations involving BRD4 or BRD3 (NCT03936465). The emerging data from such studies may identify BET inhibitors as a standard of care for NC patients in the future.

A recent drug screen identified the selective p300 histone acetylation domain inhibitor A-485, which displayed synergistic effects with BET inhibitors (55). These preclinical findings provide a strong rationale for clinical studies employing selective p300 histone acetylation domain inhibitors in patients with NC.

Furthermore, another preclinical study recently demonstrated that a distinct miRNA (miR-766-5p) targets BRD4, which can lead to the mitigation of the protumorigenic consequences of oncogenic fusion proteins, especially in the context of super-enhancers (SEs), defined as the clusters of transcription enhancers that drive gene expression. Notably, miR-766-5p was found to suppress the expression of a BRD4-NUT fusion protein that drives NC (56).

Other reports describe the use of immune checkpoint inhibitors (ICIs) in individual patients with NC (57, 58). In one study, five patients exhibiting primary pulmonary NC received ICIs as second- or higher-line treatments. An OS of 4.1 months (range, 1.5–26.7 months) was reported (58). In another recent report, two patients with NC both responded to second-line therapy with ICIs, with one patient displaying a partial response and the other a near-complete response. However, both responses were not durable and disease progression was rapid (57). Interestingly, both cases were PD-L1 negative. A major shortcoming of these studies is that patients received ICIs in a second-line setting, or beyond, following intensive chemotherapy. Thus, this approach was limited severely by factors such as (i) high tumor burden and (ii) highly immunosuppressive milieus being quite typical for advanced-stage tumor diseases. The immunotherapy drugs being applied included both PD-1 inhibitors (such as pembrolizumab and nivolumab) and PD-L1 inhibitors (such as atezolizumab) (59). While NC patients might benefit from treatment with ICIs, especially in the early stages of their tumor disease, this approach needs a more systematic research and prospective clinical trial data to testify.

## Discussion and Outlook

NC is currently a fatal disease with a poor prognosis and no effective therapeutic options. The number of reported cases is increasing; however, it is apparent that NC is still largely underdiagnosed and under-reported; thus, the true prevalence may be much higher than the numbers currently reported. With greater awareness and more systematic testing (including NGS, IHC, FISH, and PCR), NC may be diagnosed more frequently and will support the development and comparison of potential treatment strategies.

Although there are no specific pathological characteristics associated with NC, there are typical clinical and histopathological features that are indicative of NC and of which clinicians should be aware in order to diagnose patients in a timely manner. The diagnosis itself can easily be performed by demonstrating the presence of the NUTM1 antigen using IHC, and associated cut-off criteria, with a commercially available antibody to NUTM1. Because NC is a rare malignancy and patient numbers are small, every patient that presents with features suggestive of NC should be tested to reach a differential diagnosis and should be given the opportunity to participate in an ongoing clinical trial.

Major questions still needed to be answered including: (i) what cell type do NC tumors originate from (e.g., from cells exhibiting a germ cell phenotype)? (ii) How can the high proliferating capacity of NC cells be most effectively inhibited/controlled? To date, targeted agents, which have generally been applied in a monotherapeutic context, do not seem to be effective enough. Nevertheless, other novel compounds, such as BI 894999, are currently being investigated. BI 894999 is a pan-BETi inhibitor, which inhibits the binding of the BRD4-BD1 and BRD4-BD2 bromo-domains to acetylated histones with the IC_50_s of 5 ± 3 nM and 41 ± 30 nM, respectively (60). Thus, beyond the *de novo* initiation of most advanced cancer drug screens, combinatorial inhibitor approaches should find their way into clinical testing.

At present, first-line immunotherapeutic approaches have not been extensively studied in patients with NC. Immunotherapy in later-line settings is likely to be less effective due to the immunosuppressive effects of prior aggressive chemotherapeutic regimens as well as the low immunogenicity of advanced tumors. Potentially, immunotherapeutic agents could be combined with anti-proliferative agents in a first-line setting, when developing tumors might be more immunogenic. Such an approach, in conjunction with biopsy-guided and non-invasive PET-guided (e.g., employing anti-CD8 nanobodies) monitoring of NC tumors to assess infiltration of immune cells, could ultimately help achieve long-term remissions.

## Author Contributions

UL: conceptualization, data curation, formal analysis, investigation, methodology, visualization, roles/writing—original draft, and writing—review and editing. MHi: roles/writing—original draft, writing—review and editing, and resources. MHo: roles/writing—original draft and writing—review and editing. PO: roles/writing—original draft, formal analysis, and writing—review and editing. LZ: project administration, writing—review and editing, and supervision. All authors contributed to the article and approved the submitted version.

## Funding

Medical writing support was funded by Boehringer Ingelheim Pharma GmbH & Co. KG, Ingelheim am Rhein, Germany.

## Conflict of Interest

The authors declare that the research was conducted in the absence of any commercial or financial relationships that could be construed as a potential conflict of interest.

The Tübingen University Hospital receives compensation for contributions to the ongoing NUT carcinoma study (NCT02516553), sponsored by Boehringer Ingelheim Pharma GmbH & Co. KG. Medical writing support for the development of this manuscript was provided by Physicians World Europe GmbH (Mannheim, Germany), funded by Boehringer Ingelheim Pharma GmbH & Co. KG.

## Publisher’s Note

All claims expressed in this article are solely those of the authors and do not necessarily represent those of their affiliated organizations, or those of the publisher, the editors and the reviewers. Any product that may be evaluated in this article, or claim that may be made by its manufacturer, is not guaranteed or endorsed by the publisher.
