# Supplementary material for: NUT Carcinoma—An Underdiagnosed Malignancy
Source: Front Oncol. 2022 Jul 26;12:914031. doi: 10.3389/fonc.2022.914031 (PMC9360329; doi:10.3389/fonc.2022.914031)

**Supplementary material**

**Supplementary Figure 1.** Information on *NUTM1* fusion partner for 167 out of 310 cases. Information on the fusion partner for NUT was available for 149 cases. 73% of cases were *BRD4-NUTM1*, 5% were *BRD3-NUTM*, 5% were *NSD3-NUTM1*, 5% were *CIC-NUTM1*, 5% were Variant-*NUTM1*.


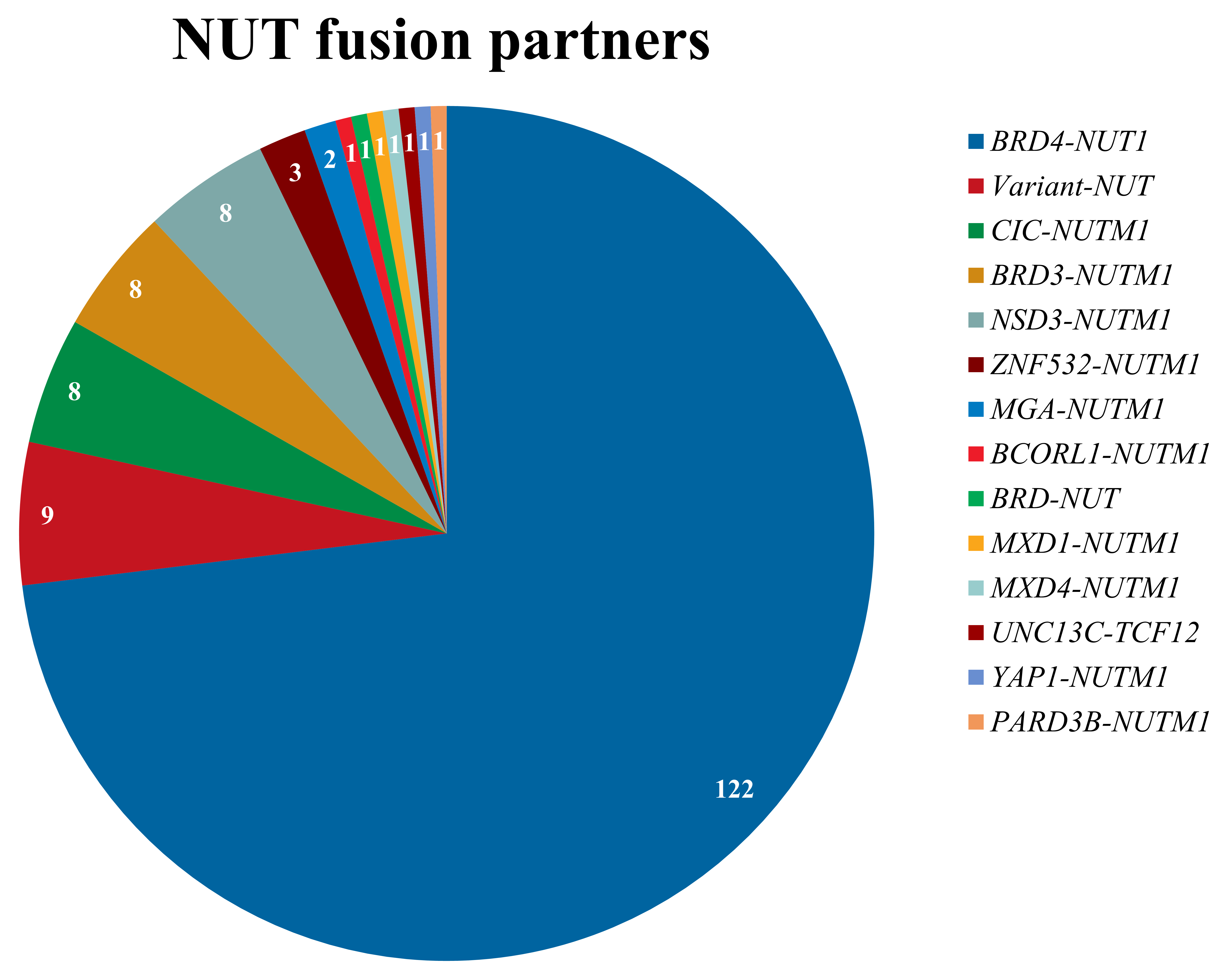


*Methodology and results of structured literature screening*

A structured literature search was performed on 20 Jan 2022 using the databases PubMed, PMC, Embase, Biosis, Northern Lights and Google Scholar (**Figure 1**). Search terms included ‘NUT carcinoma’, ‘NUT midline carcinoma’, ‘NUT rearrangement’, ‘nuclear protein in testis’ and ‘NUTM1’. For a full list of database search terms please refer to **Supplementary Table 1**. The structured search returned 336 articles for review. All abstracts were reviewed for relevance, using additional search terms screening for patient data (see **Supplementary Table 1**)**.** Upon review of the abstracts, 125 articles were discarded as irrelevant, while the in-depth review of publications revealed 12 additional relevant articles. In total, 223 articles were reviewed in detail. 48 of those were discarded on closer inspection as they turned out to be irrelevant, did not provide sufficient data on patients or presented cases that were duplicates of other publications. The remaining 175 articles identified 310 unique cases of NUT carcinoma that were analyzed further.

**Supplementary Table 1.** Search terms applied to the structured literature search for the identification of reported NUT carcinoma cases.

| Search terms for database searches | Search terms for abstract screening |
| --- | --- |
| NUT carcinoma | Case |
| NUT carcinomas | Cases |
| NUT midline carcinoma | Case report |
| NUT midline carcinomas | Case reports |
| NUT-C | Case series |
| NUT cancer | Patient |
| NUT cancers | Patients |
| NUT tumor | Male |
| NUT tumors | Female |
| NUT tumour | Man |
| NUT tumours | Woman |
| NUT rearranged | Child |
| NUT-rearranged | Children |
| NUT rearrangement | Middle Aged |
| NUT-rearrangement | Young Adult |
| Nuclear protein in testis | Adolescent |
| Nuclear protein of the testis | Teen |
| NUTM1 protein | Teenager |
| NUT protein | year old |
| NUT- | year old |
| NUT-midline | Boy |
| NUT-midline carcinoma | Girl |
| NUT |  |
| (NUT) |  |
| NMC |  |
| (NMC) |  |
| “nut” carcinoma |  |
| “NUT” |  |
| /NUT |  |
| NUT. |  |
| NUT, |  |
| NUTM1 |  |
| NUTM |  |
| NSD3-NUT |  |
| NSD3-NUT Fusion |  |
| NUT-variant midline carcinoma |  |

**Supplementary Figure 2.** Outcome of cases at time of publication broken down by disease stage at diagnosis. Outcome was recorded for 59/65 localized cases, 42/48 locally advanced cases and 68/77 metastatic cases.


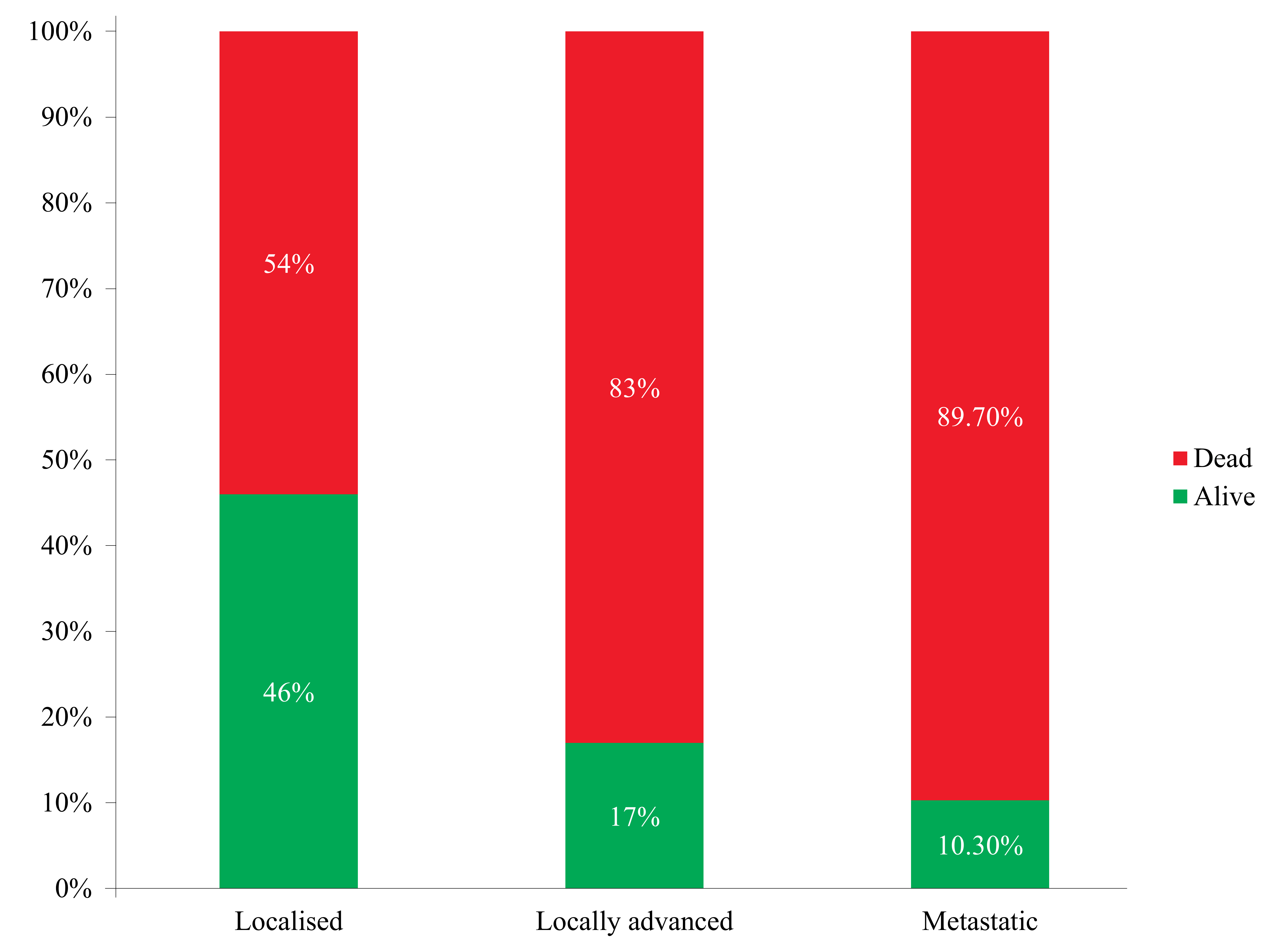

Supplement: Supplementary file 1 [file DataSheet_1.docx]
